# The economy of pollen dispersal in flowering plants

**DOI:** 10.1098/rspb.2023.1148

**Published:** 2023-10-04

**Authors:** Steven D. Johnson, Lawrence D. Harder

**Affiliations:** ^1^ Centre for Functional Biodiversity, School of Life Sciences, University of KwaZulu-Natal Pietermaritzburg, Private Bag X01, Scottsville 3209, South Africa; ^2^ Department of Biological Sciences, University of Calgary, Calgary, Alberta, Canada T2N 1N4

**Keywords:** pollen export, pollen removal, pollen transfer efficiency, pollen fates, pollinaria, siring success

## Abstract

Mating success of flowering plants depends strongly on the efficiencies of pollen removal from flowers and its subsequent dispersal to conspecific stigmas. We characterized the economy of pollen dispersal in flowering plants by analysing pollen fates and their correlates for 228 species. The mean percentage of pollen removed from flowers (removal efficiency) varied almost twofold according to the type of pollen-dispersal unit, from less than 45% for orchids and milkweeds with solid pollinia, to greater than 80% for species with granular monads or sectile (segmented) pollinia. The mean percentage of removed pollen reaching stigmas (pollen transfer efficiency, PTE) varied from 2.4% for species with separate monads to 27.0% for orchids with solid pollinia. These values tended to be higher in plants with single pollinator species and in those with non-grooming pollinators. Nectar production increased removal efficiency, but did not influence PTE. Among types of pollen-dispersal units, the net percentage of produced pollen that was dispersed to stigmas varied negatively with removal efficiency and positively with PTE, indicating the relative importance of the latter for overall pollen economy. These findings confirm the key importance of floral traits, particularly pollen packaging, for pollen dispersal outcomes and highlight the under-appreciated pollination efficiency of non-grooming pollinators.

## 1. Introduction

Mating of most flowering plants involves co-option of suitable animal vectors that transfer pollen to stigmas of conspecifics [1]. This generates pollen economy, the interrelated production, distribution and consumption activities that determine the fate of a plant's investment in male function [2–4]. In general, a plant's pollen export is the product of the total amount of pollen removed and the proportion of that pollen that vectors transport to stigmas (pollen transfer efficiency). In animal-pollinated plants, pollen removal varies with the frequency of animal visits, their behaviour, and the mechanics of pollen transfer onto the bodies of individual pollinators [5–7]. By contrast, pollen transfer efficiency depends on the extent to which removed pollen remains in transit long enough to be exchanged from the vector onto a conspecific stigma, rather than being lost to another fate [8–12]. As most produced pollen fails to reach conspecific stigmas [8], plants rarely experience both thorough removal and efficient transport of pollen [11,13,14]. The resulting pervasive limitation of pollen export is an important aspect of the context in which sexual selection through male function [15–19] should favour traits that promote siring success.

Manipulation of pollen vectors by means of floral traits is one obvious means of enhancing pollen removal and dispersal [1,20]. Animal pollen vectors act in their own interests, seeking benefits such as food, mates and shelter, while also incidentally transferring pollen among flowers. Plants encourage this searching behaviour by signalling the location of possible benefits (e.g. via displays of showy, scented flowers) and usually provide rewards to animals, such as nectar and pollen, that reinforce their searching behaviour [1]. Plant species that rely on deceit, rather than providing rewards, generally receive fewer visits and experience less pollen removal and deposition than do rewarding species [21–23], but in some cases, such as sexual deception, deceitful species may attain pollen export advantages owing to limited pollinator sharing with other plant species [24]. Pollinator manipulation may also involve traits that influence pollinator position in relation to the plant's sexual organs (e.g. floral tubes, floral symmetry, dichogamy and herkogamy), visit duration per flower (e.g. nectar accessibility and availability) and the number of flowers visited (e.g. reward levels and display size) [1].

The success of pollen removal and dispersal can also depend on the type of animal involved [25–29]. Ideal pollinators visit flowers regularly, move among plants of the same species, and do not disturb the pollen they carry. However, few, if any, animals exhibit all these behaviours. For example, although bees (Hymenoptera, Anthophila) are common flower visitors, pollen is their sole protein source and they tend to transport much of the pollen they remove from flowers to their nests, rather than to stigmas of conspecific flowers [27,30,31]. Pollen losses are likely to be particularly severe when pollinators such as syrphid flies [32] and many beetles [33] consume pollen directly from anthers. The relative advantages of different pollinator groups for pollen transfer is considered a key factor in floral specialization [34,35]. Floral traits that restrict visitor diversity (floral filtering) reduce competition for rewards among visitors and thus increase fidelity of the target visitors for conspecific flowers [36–39]. Consequently, plant species specialized for pollination by particular animals are expected to attain higher pollen transfer efficiency [15], though to our knowledge this has not been tested directly. One drawback of specialized pollination systems could be that restriction of pollinator diversity also reduces overall flower visitation and pollen removal [40], thus lessening possible pollen-transfer advantages of pollinator specialization.

**Figure 1.**
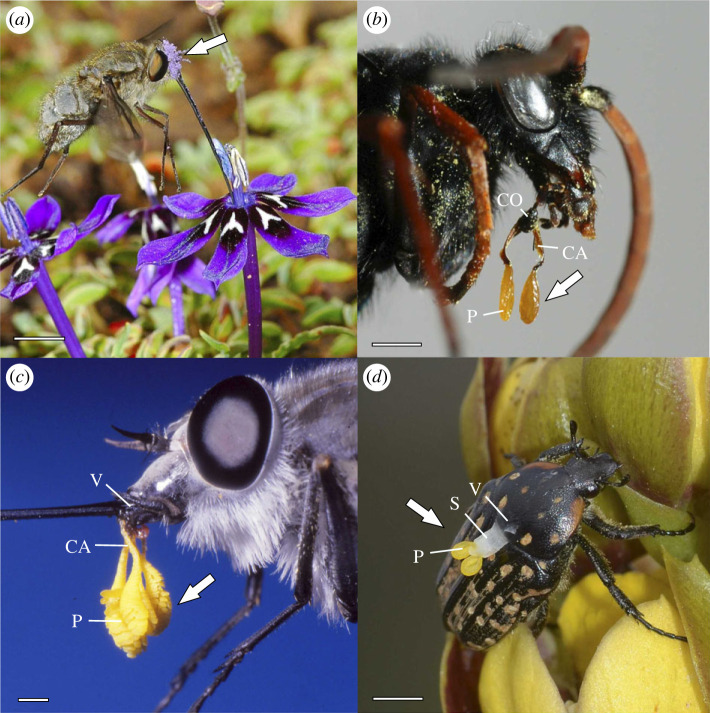
Pollen dispersal units (indicated by arrows) in flowering plants. (*a*) Granular monads of *Lapeirousia oreogena* (Iridaceae) deposited on the head of a long-proboscid fly *Prosoeca marinusi* (Nemestrinidae). (*b*) Milkweed pollinaria of *Pachycarpus asperiifolius* (Apocynaceae) clipped onto the maxillary palps of a spider-hunting wasp *Hemipesis capensis* (Pompilidae). (*c*) Sectile pollinaria of *Disa harveyana* (Orchidaceae) glued to the proboscis of a horsefly *Philoliche rostrata* (Tabanidae). (*d*) Solid pollinaria of *Eulophia parviflora* (Orchidaceae) glued to the thorax of a chafer beetle *Cyrtothrea marginalis* (Scarabaeidae). Scale bars = 10 mm. CO, corpsuculum; CA, caudicle; P, pollinia; V, viscidium; S, stipe.

A significant transition in the evolution of angiosperms involved independent shifts from dispersal of separate pollen monads to pollen packaged in pollinia (figure 1) in the Orchidaceae and Apocynaceae [41,42]. Pollen aggregations, known as pollinia, attach to pollinators via a sticky pad (viscidium) in most orchids (Epidendroideae and Orchidoideae) or a mechanical clasper (corpusculum) in milkweeds (Secamondoideae and Asclepiadoideae). Flowers with pollinia produce approximately as many individual pollen grains as do those with monads [43,44]. Solid pollinia of most orchids (approx. 80% of species [45]) and all milkweeds are deposited as an entire unit on a recipient stigma. By contrast, roughly 11% of orchid species, including most temperate species, have ‘sectile' pollinia comprised numerous segments known as massulae [45,46], each containing several hundred pollen grains [41], which enables some pollen carryover between successively visited flowers [9,47,48]. Collectively, a set of pollinia and its associated attachment device comprise a pollinarium. The firm attachment of pollinaria to pollinators has been posited to reduce pollen loss greatly during transport, but this has been tested for only a small number of species, without considering additional factors such as pollinator type and diversity [14].

This study uses a large sample of species to assess potential influences on the economy of pollen dispersal in animal-pollinated flowering plants. The examined influences include: (i) the type of pollen aggregation (granular monads, sectile and solid orchid pollinia, and solid milkweed pollinia); (ii) specialization of pollination systems, with respect to both particular pollinator functional groups (e.g. bees versus beetles) and the overall number of pollinator taxa; and (iii) production of floral nectar. In addition to illustrating the spectrum of pollen economies within angiosperms, this analysis identifies the aspects of pollen dispersal that are most likely involved in sexual selection on male mating traits.

## 2. Methods

### (a) Pollen economy metrics

Key features of a species' pollen economy such as pollen transfer efficiency (PTE) can be estimated using population means of pollen production per flower, *P*, the pollen remaining in wilted flowers, *L*, and the pollen deposited on their stigmas, *D*. Note that *D* can include both self- and cross-pollen. Mean pollen removal can be estimated as *R* = *P* – *L*. For a population in which pollen either disperses exclusively among resident plants or pollen emigration equals immigration, mean pollen export, *E,* can be estimated as

E=R×PTE,

where

2.1 $$\mathrm{PTE}=\frac{D}{R}.$$

To compare species for which different pollen units (monads, sectile pollinia and solid pollinia) were counted to quantify *P*, *L* and *D* (see below), we considered several relative metrics, including pollen removal efficiency:

$$\mathrm{RE}=\frac{R}{P},$$

and relative pollen export:

2.2 $$\mathrm{RPE}=\frac{E}{P}=\mathrm{RE}\cdot \mathrm{PTE}=\frac{/R}{P}\cdot \frac{D}{/R},$$

(equivalent to Inouye *et al*.'s [12] pollen production efficiency). Note that, unlike pollinaria for which removal requires pollinator contact in outcrossing species, granular pollen can be removed from flowers by other agents, including being dislodged by plant shaking or direct loss to wind and rain. To the extent that the absence of pollen from flowers does not represent removal by pollinators, PTE (equation (2.1)), but not RPE (equation (2.2)), will tend to be underestimated. In the remainder of the paper RE, PTE and RPE are referred to as removal, transfer and export efficiencies, respectively.

### (b) Data characteristics

We obtained data on pollen fates for 228 animal-pollinated species that provided information concerning removal efficiency, transfer efficiency or both (electronic supplementary material, S1), including records from published studies for 208 species and our own previously unpublished studies of 36 species (20 of which had not been studied previously in terms of pollen fates). Our analyses did not consider species with unusual pollen-dispersal types, such as the tetrads and the style head adhesive system of *Apocynum* (Apocynaceae) which is represented by a single species study [49]. The global coverage included 13 species from Europe, 125 from Africa, 30 from Asia, 34 from North America and 26 from South America. Data were available for too few species from Australia and New Zealand (*n* = 2) for this region to be included. For 44 species represented by data from several populations or sources, we used medians for analysis. In addition to these focal variables, we recorded the continent (region) where each study was conducted and whether a studied species produces nectar, its type of pollen-dispersal unit, its dominant pollinator functional group and the number of pollinator species, if available (electronic supplementary material, S1). For data concerning a species' pollinators, we used only focused studies (typically involving five or more days of observations) which identified animals that transported pollen. Pollinator functional groups included bees, beetles, wasps, short-proboscid flies, long-proboscid flies, lepidopterans, and birds. Short- and long-proboscid flies were distinguished owing to their marked functional differences [50]. A plant species was considered to be pollinated primarily by a particular animal functional group if that group contributed at least 75% of all flower visits likely to result in pollination [34], otherwise it was considered to have a generalist pollination system.

Our own studies (electronic supplementary material, S1), were conducted between 2003 and 2020 and involved samples of flowers from 9 to 331 (median = 54) flowers per population with a single flower sampled per plant in most cases. For individuals of species in which pollen disperses as monads, we collected the anthers of newly opened flowers to estimate pollen production, and the anthers (or pollen presenter) and stigmas of wilted flowers to quantify unremoved pollen and conspecific pollen deposition, respectively. Anthers and stigmas were preserved and stored in separate microcentrifuge tubes in 70% ethanol. Pollen production was counted using either an electronic particle counter [5] or a compound microscope. Unremoved pollen and pollen grains on squashed stigmas were counted with a compound microscope after staining with basic fuchsin [51]. For milkweeds and orchids with solid pollinia, pollen economies were quantified based on pollinarium number and removal, and pollinium deposition on stigmas [52]. For orchids with sectile pollinia, we quantified the production and fates of individual massulae. To estimate the number of massulae in sectile pollinia, we dabbed individual pollinia from 5 to 17 (median = 10) flowers per species onto sticky tape and counted the adhering massulae under a dissecting microscope. Massulae on stigmas were counted with a dissecting microscope or 20× hand lens. For older flowers, counting of massulae enveloped in stigmatic fluid was aided by first dipping the entire stigma in a 10% solution of methylene blue stain.

### (c) Statistical analyses

All analyses involved generalized linear models [53] as implemented in the glimmix procedure of SAS/STAT 15.2 [54]. These analyses considered beta distributions and involved the logit link function, as all dependent variables are proportions (proportion of pollen removed, PTE, relative pollen export). The beta distribution is undefined for a proportion of 1, so we replaced such values with 1 – 1/(2·*f*) for species for which the number of flowers sampled, *f*, was known. These analyses did not account for phylogenetic relatedness among species because the examined pollination outcomes resulted from ecological interactions with pollinators [55], rather than being genetically determined traits that are direct targets of natural selection. We checked this assumption for each analysis by quantifying phylogenetic signal (Blomberg's K [56]) for the residuals from the analysis, as described in the electronic supplementary material, appendix S1. No statistical evidence of phylogenetic signal was detected for any analysis (electronic supplementary material, tables S1 and S2).

Most analyses considered pollen dispersal unit (monad, massula, solid orchid pollinium, milkweed pollinium), whether a species produced nectar, and its dominant pollinator functional type (defined above) as categorical independent variables. We also considered whether pollinators consume pollen and/or groom frequently, as these behaviours are expected to increase transport loss [27,31]. This variable involved two categories: grooming—frequent pollen consumption/grooming, which included species pollinated by beetles, short-tongued flies, bees, or combinations of these insects; or non-grooming—infrequent grooming, including species pollinated by long-tongued flies, wasps, lepidopterans, or birds. Some analyses of transfer and export efficiency also included removal efficiency as a continuous covariate. We also assessed the effects of pollinator diversity (one versus many species) on each dependent variable for the subset of species for which relevant information was available. The latter two analyses also included pollen type, nectar production and either pollinator type or grooming, as described above; however, interpretation of them focused on pollinator diversity. The analyses of transfer efficiency also accounted for heterogeneous variances in the dependent variable among types of pollen dispersal units. For the marginal means of categorical effects, *post hoc* analysis of main effects involved Tukey's tests, whereas that of interactions and nested effects involved Dunn-Šidák comparisons [57]. For the overall analyses of transfer and export efficiency, we also considered whether the means for the different types of pollen dispersal units varied linearly with the marginal dispersal-unit mean logits for removal and transfer efficiency (analysis of export efficiency only) estimated by their respective analyses (i.e. linear trend analysis: [57]). In addition to the focal independent variables described above, all analyses accounted for possible effects of the region where each source study was conducted. All analyses initially included all possible interactions between the independent variables, which could be excluded by backward elimination during subsequent analysis. This approach guarded against the consequences of collinearity between independent variables. The main effects of all independent variables hypothesized to affect pollen economies were included in all final models. We graphically present marginal effects of independent variables, holding the effects of other independent variables constant. For categorical effects, we present marginal (least squares) means and 95% confidence intervals (CI) [58]. For the effect of continuous variation in the pollen-removal proportion on transfer efficiency, we adjusted observations by adding an observation's residual to its expected value predicted by the marginal regression equation.

## 3. Results

### (a) Pollen removal

**Figure 2.**
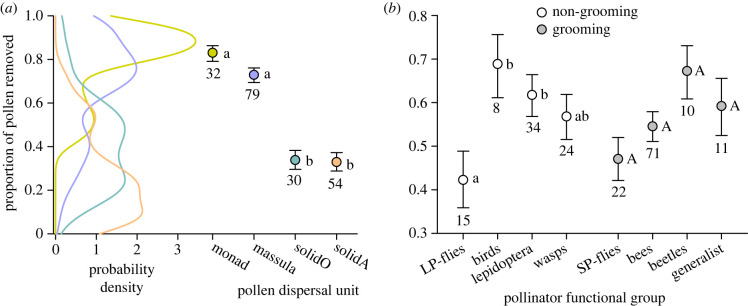
Interspecific variation in removal efficiency (*a*) within and among types of pollen dispersal unit and (*b*) among functional groups of non-grooming and grooming pollinators. In (*a*), the curves depict the probability density distribution of removal efficiency for each type of dispersal unit. Panels (*a*) and (*b*) illustrate marginal means (± s.e.): means that share the same letter do not differ statistically (a, Tukey's test; b, Dunn-Šidák comparisons). In (*b*), lower- and upper-case letters distinguish the separate Dunn-Šidák comparisons applied within the two grooming categories. Numbers adjacent to error bars indicate the number of species per group. SolidO and solidA refer to the solid pollinia of orchids and milkweeds, respectively. See the electronic supplementary material, table S1 for statistical details.

The average proportion of pollen removed from flowers varied statistically among types of pollen dispersal units (hereafter ‘dispersal types'), pollinator types and region, but not with whether the species produced nectar or its dominant pollinators groom frequently (electronic supplementary material, table S1). Although proportional pollen removal varied extensively among species within dispersal types, the mean removal efficiency for species with solid pollinia (orchids and milkweeds) was generally half that of species with separate monads or massulae ($\chi_{1}^{2}=131.4$, *p* < 0.001; figure 2*a*). Mean removal efficiency differed among species pollinated by different functional groups of non-grooming pollinators ($\chi_{3}^{2}=9.78$, *p* < 0.05), but not for those served by grooming pollinators ($\chi_{3}^{2}=7.53$, *p* > 0.05; figure 2*b*). The statistical difference among species with non-grooming pollinators specifically involved proportionally less pollen removal by long-proboscid flies than by birds or Lepidoptera (figure 2*b*). For a subset of 152 species with relevant information, removal efficiency also did not differ among species served by a single versus multiple pollinators (electronic supplementary material, table S1). For both analyses, removal efficiency also varied among geographical regions (electronic supplementary material, table S1 and appendix S2, figure S1*a*).

### (b) Pollen transfer efficiency

**Figure 3.**
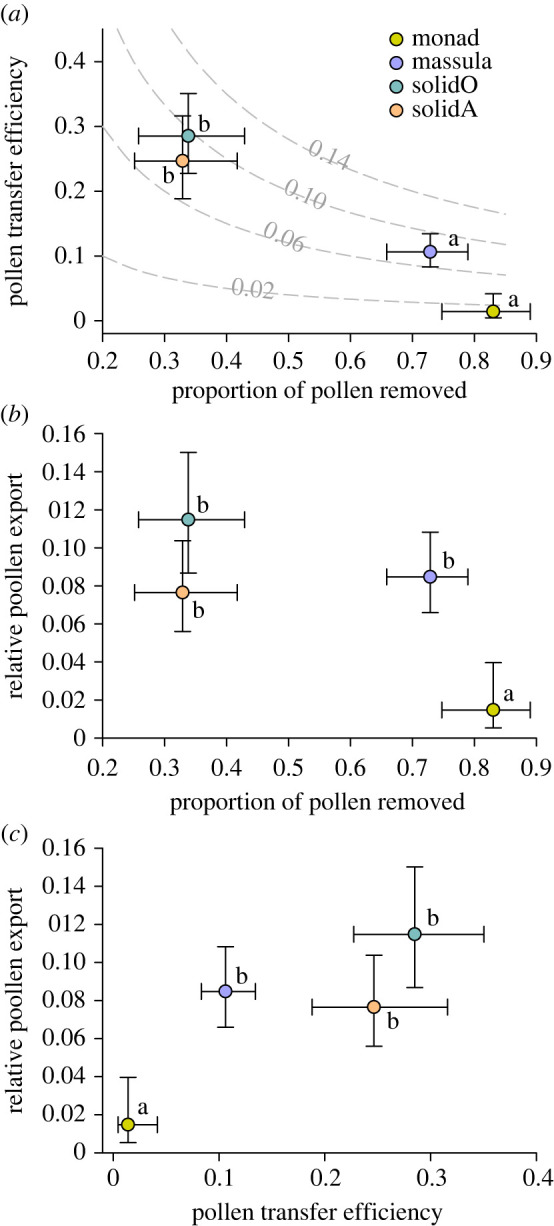
Associations of (*a*) mean pollen transfer efficiency (PTE) with mean pollen removal efficiency (RE) and of mean relative pollen export (RPE) with (*b*) mean RE and (*c*) mean PTE for different types of pollen dispersal unit. All pairs are marginal means (± 95% CI) are drawn from separate analyses of the respective variables. Letters identify statistical differences among dispersal types for the variable identified on the ordinate: means that share the same letter do not differ statistically (Tukey's test). SolidO and solidA refer to the solid pollinia of orchids and milkweeds, respectively. Isoclines in (*a*) indicate the overall proportion of produced pollen that reaches stigmas (specifically, relative export efficiency = 0.02, 0.06, 0.10, or 0.14) for different combinations of RE and PTE, based on (equation (2.2)).

Overall, marginal mean transfer efficiency varied more than an order of magnitude among dispersal types, from 1.4% for species with monads to 28.5% for orchids with solid pollinia (electronic supplementary material, table S2 first analysis; figure 3*a*). Mean transfer efficiency generally varied negatively with mean removal efficiency among dispersal types (figure 3*b*: linear trend, $\chi_{1}^{2}=44.30$, *p* < 0.001). Specifically, milkweeds and orchids with solid pollinia experienced limited removal but high transfer efficiency, whereas species with massulae and separate pollen monads experienced extensive removal but low transfer efficiency. Interspecific variance in transfer efficiency differed among dispersal types (electronic supplementary material, table S2) primarily because of greater variance among species with solid pollinia than among those with massulae or monads ($\chi_{1}^{2}=13.64$, *p* < 0.001).

**Figure 4.**
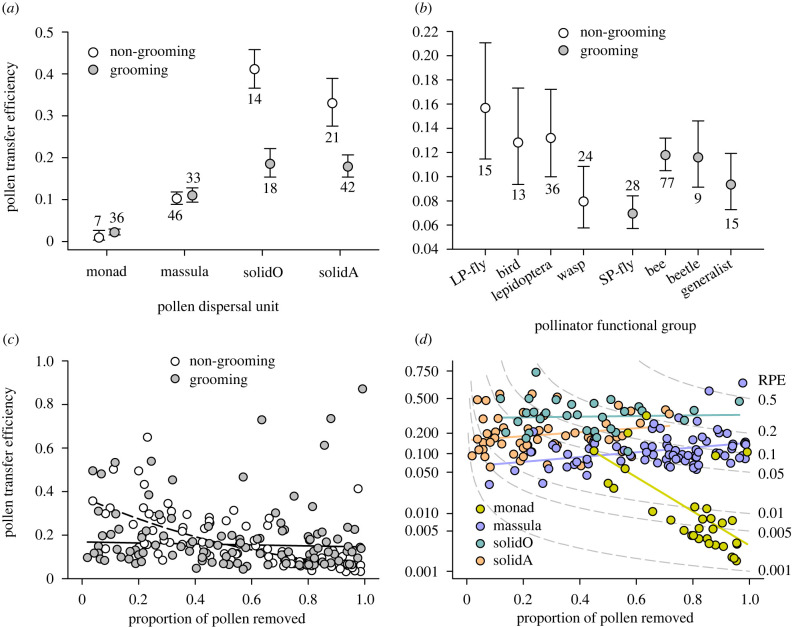
Effects on pollen transfer efficiency (PTE), including a species': (*a*,*d*) type of pollen dispersal unit, (*a*–*c*) whether its dominant pollinators groom, (*b*) the functional class of its dominant pollinator and (*c*,*d*) its pollen removal efficiency (RE). Panels (*a*) and (*b*) depict marginal means (± s.e.) and the numbers of sampled species are indicated. In (*d*), solidO and solidA refer to the solid pollinia of orchids and milkweeds, respectively. Isoclines in (*d*) indicate the overall proportion of produced pollen that reaches stigmas (relative export efficiency; RPE) for different combinations of removal efficiency and transfer efficiency, based on (equation (2.2)). For (*d*), note the logit scaling of the ordinate.

Transfer efficiency also varied among species according to whether their dominant pollinator grooms frequently (figure 4*a*), but not the pollinator's particular functional group (figure 4*b*) or whether the species produces nectar (electronic supplementary material, table S2 first analysis). The grooming effect involved only milkweeds and orchids with solid pollinia, with higher transfer efficiency for species with non-grooming pollinators (figure 4*a*: orchids, $\chi_{1}^{2}=15.78$, *p* < 0.001; milkweeds, $\chi_{1}^{2}=8.77$, *p* < 0.025: based on Dunn-Šidák comparisons). An analysis of the subset of species with records of pollinator diversity found similar transfer efficiency for species with a single or multiple pollinator species (electronic supplementary material, table S2 second analysis). For both analyses, transfer efficiency also varied among geographical regions (electronic supplementary material, table S2 and appendix S2, figure S1*b*).

An additional analysis that considered the effects of removal efficiency on transfer efficiency detected separate interactions of removal efficiency with whether the dominant pollinators groom and pollen dispersal type (figure 4*c*,*d*; electronic supplementary material, table S2 third analysis). Transfer efficiency varied negatively with removal efficiency for species with non-grooming pollinators (partial regression coefficient, *b* ± s.e. = −2.300 ± 0.555; $\chi_{1}^{2}=17.17$, *p* < 0.001), but not for those with grooming pollinators (−0.178 ± 0.488; $\chi_{1}^{2}=0.13$, *p* > 0.7). This contrast indicates that non-grooming pollinators elevated transfer efficiency for species subject to limited pollen removal (mostly milkweeds and orchids with solid pollinia), but reduced transfer efficiency for species with extensive removal (mostly species with monads or massulae) compared to species with grooming pollinators (figure 4*c*). The relationship of transfer efficiency to removal efficiency also differed among pollen types, being neutral for species with solid pollinia (milkweeds – *b* ± s.e. = 0.797 ± 0.615; $\chi_{1}^{2}=1.68$, *p* > 0.1: orchids – 0.128 ± 0.683; $\chi_{1}^{2}=0.04$ , *p* > 0.8); weakly positive for orchids with massulae (0.954 ± 0.402; $\chi_{1}^{2}=5.63$, *p* < 0.025), and strongly negative for species with separate pollen monads (−6.836 ± 1.555; $\chi_{1}^{2}=19.31$, *p* < 0.001; figure 4*d*). This analysis also accounted for heterogeneous overall variation in transfer efficiency among dispersal types and the influences of nectar production and geographical regions (electronic supplementary material, table S2 and appendix S2, figure S1*b*).

### (c) Relative pollen export

The marginal mean proportion of produced pollen that reached stigmas (relative pollen export) differed among dispersal types, with orchids with solid pollinia exporting a 7.8 times greater proportion of their produced pollen than species with monads (figure 3*b,c*). Given that export efficiency is the product of removal efficiency and transfer efficiency (equation (2.2)), export efficiency might be expected to vary positively with both its components. In keeping with this expectation, mean export efficiency varied positively among the different dispersal types in association with their mean transfer efficiency (figure 3*c*: linear trend; $\chi_{1}^{2}=14.74$, *p* < 0.001). By contrast, mean export efficiency varied negatively with mean removal efficiency (figure 3*b*: linear trend; $\chi_{1}^{2}=13.75$, *p* < 0.001), as did transfer efficiency (figure 3*a*).

**Figure 5.**
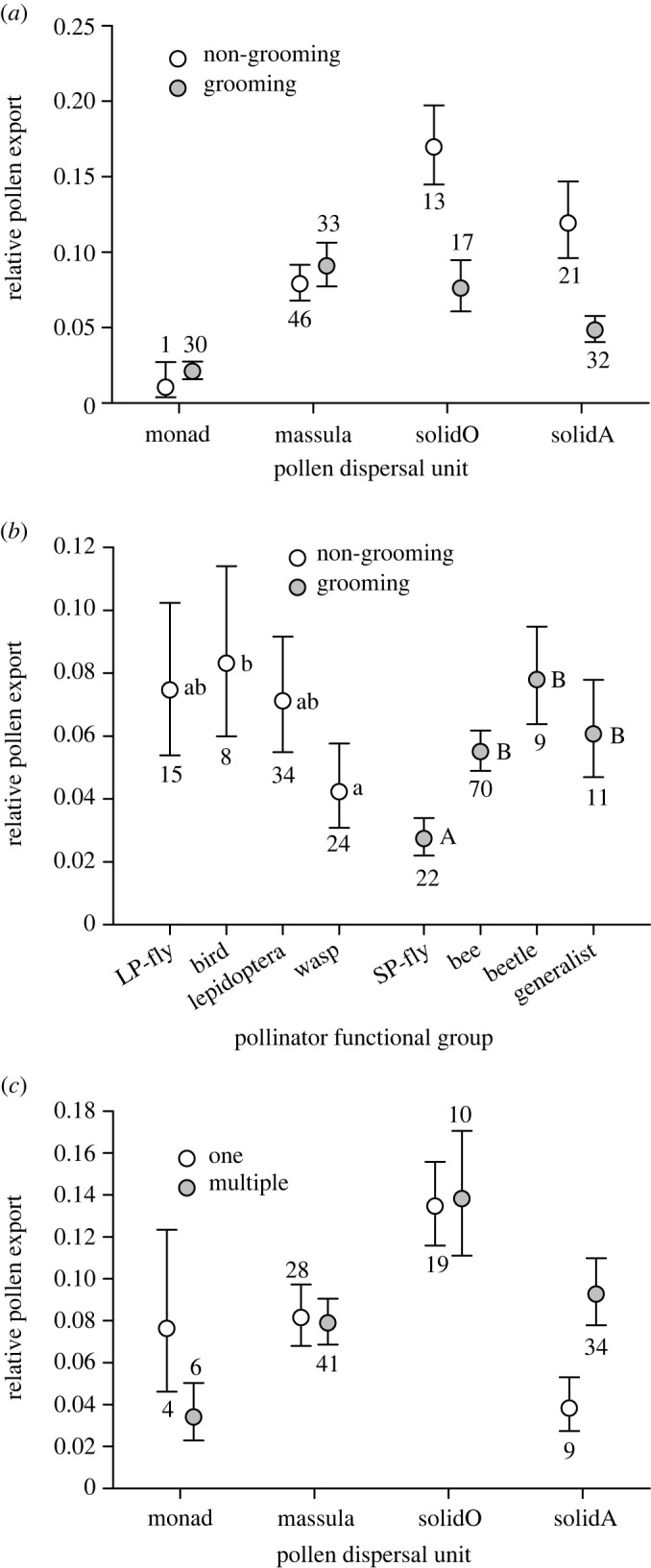
Associations of marginal mean (± s.e.) relative pollen export with a species’: (*a*,*c*) type of pollen dispersal unit, (*a*,*b*) whether its dominant pollinators groom, (*b*) the functional class of its dominant pollinator, and (*c*) whether it is primarily pollinated by one or multiple species. In (*b*), lower- and upper-case letters distinguish the separate Dunn-Šidák comparisons applied within the two grooming categories. Numbers adjacent to error bars indicate the number of sampled species per group.

Pollen export efficiency additionally varied among species in association with several characteristics of their dominant pollinators (figure 5; electronic supplementary material, table S2). Differences in mean export efficiency varied depending on whether dominant pollinators groom frequently for species with solid pollinia (orchids, $\chi_{1}^{2}=13.76$, *p* < 0.001; milkweeds, $\chi_{1}^{2}=21.76$, *p* < 0.001: based on Dunn-Šidák comparisons), but not for species with monads or massulae (dispersal unit×grooming interaction). Among species with solid pollinia, those with non-grooming pollinators experienced greater export efficiency than for species with grooming pollinators (figure 5*a*). Export efficiency also differed among species with different pollinator functional groups (figure 5*b*). Among species with non-grooming pollinators, wasp-pollinated species experienced lower export efficiency than bird-pollinated species. Additionally, species pollinated by short-proboscid flies realized lower export efficiency than species with other grooming pollinators, including generalist pollination. A separate analysis for species with records of pollinator diversity detected lower export efficiency for milkweeds with a single pollinator species than for those with multiple pollinators ($\chi_{1}^{2}=8.67$, *p* < 0.025), but no diversity effect among species with other dispersal types (all other Dunn-Šidák comparisons *p* > 0.1: figure 5*c*; electronic supplementary material, table S2 [second analysis]). The analysis that included diversity suggested the only evidence of differences related to whether species produce nectar, at least among South American species (electronic supplementary material, table S2, appendix S2, figure S1d). The generality of this result is uncertain, as it involved exceptionally high marginal mean export efficiency for the two nectarless species in comparison with 24 nectar-producing species. Both analyses also accounted for other influences of geographical regions (electronic supplementary material, table S2, appendix S2, figure S1).

## 4. Discussion

Pollen dispersal is a highly uncertain process with extremely variable outcomes in terms of the proportion of pollen produced by plants that reaches conspecific stigmas (relative pollen export; figures 3 and 5: also see [8,9,59]). Given that pollen export efficiency is the product of removal and transfer efficiencies (equation (2.2)), it might be expected to vary positively with both of its components. In keeping with this expectation, mean export efficiency for the different dispersal types varied positively with their mean transfer efficiency (figure 3*c*). By contrast, mean export efficiency varied negatively with mean removal efficiency (figure 3*b*), as did transfer efficiency (figure 3*a*). That export efficiency varies as expected with transfer efficiency but not with removal efficiency indicates that variation in export efficiency arises primarily from differences among species in the success of pollen transfer to stigmas, rather than the success of pollen removal from anthers. Variation in pollen economy among species also relates to consequences of whether pollen is dispersed to conspecific stigmas as separate monads, massulae of sectile pollinia, or solid pollinia (figures 3 and 5*a–c*). By comparison, nectar production and pollinator type, diversity and grooming propensity had more limited effects on pollen removal efficiency (figure 2*b*), transfer efficiency (figure 4) and export efficiency (figure 5).

### (a) Pollen removal

The proportion of pollen removed from flowers depends on the efficiency of removal per pollinator visit and the number of visits per flower, both of which are linked to dispersal type. For species with monads, pollen can be physically dislodged from anthers by wind and rain or be removed by pollen thieves [27], so removal efficiency may not fully represent the potential for their pollen to be dispersed to stigmas. Nevertheless, the proportion of removed monads with dispersal potential may be relatively high, as estimates from broad surveys of species with monads indicate a mean of approximately one visit per flower per hour [60,61], though this is highly variable among species and regions [62]. Milkweeds also often experience frequent visitation [25,63,64], but their removal efficiency per visit is often low [25] because the mechanical clipping system of their corpuscula requires very precise contact with a suitable pollinator structure [65,66]. Furthermore, each corpusculum of the five pollinaria must be contacted separately for complete removal of a milkweed flower's pollen. By contrast, pollen-removal failure of orchids probably arises primarily from low visit frequency, as a single visit can remove a flower's entire pollen complement. Low visit frequency, in turn, may reflect the low-density populations and rewardlessness of many orchid species [22,23]; however, we did not detect specific effects of nectarlessness on removal efficiency for orchids perhaps owing to their effective strategies of deception, including mimicry [21].

### (b) Pollen transfer efficiency

Less than half the pollen removed from flowers reached conspecific stigmas (figure 3*a*,*d*), indicating pervasive transport loss. This loss arises from diverse causes, including pollen not adhering firmly to pollinator bodies, being displaced [48,67], grooming and consumption by pollinators (including transport to bees' nests [27,68–70]), over-layering on pollinators' bodies by newly added pollen [48,71,72] and pollen deposition on stigmas of other species owing to pollinator inconstancy [73]. As low transfer efficiency is universal, it should select strongly for traits that limit these causes of transport loss.

Differences among species in dispersal type account for much interspecific variation in transfer efficiency (figures 3*a* and 4*a*). The pattern of differences in mean transfer efficiency among dispersal types probably indicates the combined influences of attachment mechanisms and pollen carryover properties. Most generally, species with pollinaria attain higher transfer efficiency than those with monads because pollinaria are glued (orchids) or clipped (milkweeds) onto the bodies of pollinators, reducing transport loss. Additional variation among dispersal types probably reflects patterns of pollen carryover, in particular the minimal number of flower visits needed to deplete the pollen carried by a pollinator as it moves from individual source flowers to multiple recipients, because longer persistence should increase the chance of transport loss. This minimal visit number ranges from one flower for many orchids with solid pollinia [41], to two flowers for milkweeds [74], to a few to 20 flowers for species with massulate pollinia [9,47,48], to tens of flowers for species with monads [59,74,75]. In general, lock and key pollination mechanisms in orchids and milkweeds [46,76] may also reduce pollen losses through heterospecific pollen transfer (but see [77]). Thus, the observed transfer efficiency variation indicates multifaceted consequences of the evolution of different types of pollen dispersal units from the ancestral monad dispersal.

Pollinator type and specialization in pollination systems were found to have only weak effects on overall pollen economy. The generally equivalent transfer efficiency among pollinator types within grooming classes (figure 4*b*) contradicts the popular perception of bees as ideal pollinators [78] and highlights the comparable effectiveness of less-appreciated pollinator groups, such as flies and beetles, for overall pollen transfer. Although pollination system specialization is considered to enhance the effectiveness of pollen dispersal in plants [79,80], it did not affect removal or transfer efficiency in our study and influenced export efficiency only for orchids with solid pollinia (figure 5*c*). These findings may shed light on the wide diversity of animal taxa that plants use as pollinators [81], as well as the circumstances leading to switches among pollinators. If pollinator groups do not differ markedly in their effectiveness as agents of pollen transfer, then shifts among pollinators during angiosperm evolution may ultimately have more to do with the local abundance of potential pollinators and the trait modifications required to exploit them than with differences in their intrinsic effectiveness for pollination.

We also found that grooming by pollinators affected mean transfer efficiency, although this effect is conditional on the type of pollen-dispersal unit and the extent of pollen removal (figure 4*a*,*c*). Specifically, non-grooming pollinators provide somewhat elevated transfer efficiency for species that also experience limited pollen removal (figure 4*c*), many of which have solid pollinia (figure 2*a*). This result indicates that, despite the firm attachment of their pollinaria to pollinators, species with solid pollinia are not immune to grooming losses. Equally surprising is the statistically greater mean transfer efficiency for species with grooming pollinators and high pollen removal (figure 4*c*), most of which have separate monads or massulae (figure 2*a*). This difference is a consequence of transfer efficiency varying independently of removal efficiency for species with grooming pollinators (figure 4*c*), which contrasts with the negative relationship expected mathematically because absolute pollen removal is the denominator of transfer efficiency (equation (2.1)). Given the negative expectation, the observed independence requires offsetting enhancement of mean pollen dispersal (transfer efficiency numerator) with increasing total removal for species served by grooming pollinators. This positive effect could reflect adaptation to diminishing increases in total export with increased per-visitor removal [13]. Diminishing returns can arise if pollinators groom more frequently or intensively after removing abundant pollen from a flower or plant [31], aggravating transport loss. Relevant adaptation involves diverse traits that restrict per-visit pollen removal and attract more pollinators [13,82]. Optimal restriction of per-visit removal given abundant visitation can enhance pollen export by an order of magnitude, depending on the severity of the diminishing returns [82]. This explanation suggests a link between removal and transfer efficiency enhancement with trait diversification for at least those species served by grooming pollinators.

An important caveat of transfer efficiency as a measure of pollen economy is that it weights conspecific pollen on stigmas equally, even though pollinators can vary in the quality of pollen that they deposit on stigmas [83]. For the majority of the approximately 37% of angiosperm species that are self-incompatible [84], self-pollination contributes no more to siring success than pollen lost during transport; whereas for species that are self-compatible or have late-acting self-incompatibility, self-pollination can be associated with detrimental effects on ovule fertilization and seed development [85–87], reducing both female and male success. Similar qualifications apply to biparental inbreeding. Additional aspects of pollen quality that are not evident from transfer efficiency arise from possibly beneficial effects as a male and female parent from outcrossing with multiple partners [88]. As the extents of uni- and bi-parental inbreeding and outcross vary extensively among species [23,88,89], the transfer efficiency patterns that we detected probably expose an incomplete perspective on effective pollen export among plants within populations. Given the well-documented disadvantages of self-pollination for offspring quality and pollen and ovule discounting and possibly of low-diversity outcrossing, it is important to reflect on whether traits such as pollinia that enhance transfer efficiency may also involve costs that are not represented in our measures of pollen economy. For example, as pollen packaged in pollinia is expected to have limited carryover, plants with pollinia, particularly solid ones, may experience more geitonogamous self-pollination. Indeed, it has been argued that non-rewarding flowers and traits such as pollinarium bending mechanisms have been selected in orchids to ameliorate geitonogamy [90,91]. The extent to which geitonogamy limits fitness of orchids and milkweeds remains unclear, but it seems reasonable that it plays a role. However, available estimates for orchids with sectile pollinia indicate geitonogamy comparable to that of species with granular pollen [9], suggesting that the transfer efficiency advantage of sectile pollinia also applies to the cross-pollen component.

### (c) Functional evolution of pollen dispersal

Consideration of the associations of pollen economy with dispersal type reveals at least three relationships that probably shape the evolution of pollen dispersal, two of which were identified by this study. First, contrary to widespread expectation (e.g. [46,92–94]) pollen economy seems to vary little among species with ovule number. For example, recent studies have demonstrated that pollen production per flower varies positively with latitude but ovule number does not [95], and that transfer efficiency varies positively with pollen production but not with ovule production [43]. Therefore, the evolution of pollen economy probably occurs largely independently of the evolution of ovule production.

Second, mean transfer efficiency varies negatively among dispersal types with their mean removal efficiency (figure 3*a*). A general negative relationship is expected (equation (2.1)); however, the association of dispersal of solid pollinia (milkweeds and orchids) with low pollen removal and high transfer efficiency, but of massula and monad dispersal with higher removal and lower transfer efficiency (figure 3*a*) was not expected *a priori*. This contrast indicates that the evolution of different pollinium types in orchids and milkweeds from ancestral monad dispersal (see [14]) was owing primarily to selection on traits involved in pollen transport, despite generally negative consequences for pollen removal, especially for species with solid pollinia (figure 2*a*). Furthermore, the extremely low transfer efficiency among species with monad dispersal suggests strong constraints on the evolution of innovations that could reduce monad losses during transport to the extent achieved by plants with pollinaria (figure 4*d*).

The third relationship of particular interest is the strongly negative association between transfer efficiency and removal efficiency among species with separate monads, contrasting with the general independence of this relationship for species with pollinia (figure 4*d*). Harder & Thomson [13] observed that transfer efficiency of pollen removed by individual pollinators from a species with separate monads decreased as the amount of pollen removed from donor flowers increased, indicating diminish returns on increased removal. They proposed that diminishing returns should be pervasive for species with separate monads, as is evident in figure 4*d*, and that diminishing returns should select for diverse floral [13,84] and inflorescence [20] traits that restrict pollen removal per flower visitor. The implication of diminishing returns for pollen export efficiency is revealed by comparing the regression line for species with monads to the isoclines in figure 4*d*. Each isocline depicts the set of values of transfer efficiency and removal efficiency that result in a specific value of export efficiency (see equation (2.2)). Over the range of removal efficiency observed for species with monads (0.45–0.99), mean transfer efficiency declined from 0.1 to 0.003 (figure 4*d*, yellow line). As indicated by the isoclines (figure 4*d*, grey curves) crossed by this regression line, export efficiency correspondingly declined from 0.048 to 0.003. Therefore, among species with separate monads export efficiency varies positively with transfer efficiency, but negatively with removal efficiency. For such species, the evolution of enhanced export efficiency probably involves resolving a trade-off between transfer efficiency and removal efficiency. The extensive diversity of flowers and floral displays among species with separate monads may reflect alternative optimal resolutions of this trade-off in response to contrasting pollination environments.

Turning to orchids and milkweeds, Harder & Johnson [14] proposed that, in addition to greatly reducing pollen transport losses overall, pollinia relaxed diminishing returns during export compared to species with monads. This expectation is supported by the neutral (solid pollinia) or slightly positive (massulate pollinia) relations of transfer efficiency to removal efficiency among species with the same pollinium type (figure 4*d*). These relationships reveal that, in addition to elevating mean transfer efficiency by an order of magnitude (figure 3*a*), the evolution of different pollinium types effectively eliminated removal-dependent pollen loss during transport among flowers. Furthermore, based on the relationships of the regression lines of transfer efficiency for each pollinium type to the export-efficiency isoclines (figure 4*d*), export efficiency varies strongly positively with removal efficiency for species with pollinia. Thus, improvement in export efficiency among species with the same pollinium type seems largely to have involved the evolution of floral traits that enhanced pollen removal efficiency, rather than influencing transfer efficiency.

Several features of pollinia and pollinaria probably contributed to shifts in pollen economy during orchid and milkweed evolution. Packaging pollen in pollinia renders it unsuitable as food for flower visitors [96,97], and so less susceptible to consumptive loss. Inclusion of an attachment device as a pollinarium component (orchid viscidium, milkweed corpusculum) both reduces the chances of pollen being dislodged from pollinators during transport and generally ensures that the pollen is ideally positioned to contact subsequent stigmas of subsequently visited flowers [14,41]. Finally, in contrast to the improved transfer efficiency associated with pollinia, on average, such pollen aggregation is associated with removal of relatively less of the produced pollen than is the case from species with separate monads (figure 2*a*).

The contrast of this general removal disadvantage with the transport benefits suggests that pollinia evolved in angiosperms under sexual selection for enhanced pollen transport rather than enhanced pollen removal [14]. Darwin [98] and Dressler [46] argued that the evolution of numerous ovules in orchids associated with their small seeds precipitated the evolution of pollinia because this packaging allows numerous pollen grains to be delivered simultaneously to stigmas for ovule fertilization. However, this hypothesis cannot apply in the case of pollinium evolution in milkweeds, as they produce relatively large seeds. Furthermore, Darwin and Dressler's focus on seed production (female function) ignores the importance of male function as an equally relevant fitness component (see [99]). Instead, some authors [8,41] have emphasized the siring advantages of pollinia for both pollen removal under conditions of pollinator scarcity and transfer efficiency between flowers, the latter conclusion being supported by the present study. That such pollen aggregation has evolved only twice in angiosperms indicates strong constraint on this innovation and its enhancement of transfer efficiency. Breaching this constraint during evolution in the orchid and milkweed clades was apparently facilitated by prior fusion of the androecium and gynoecium [14], which has otherwise occurred rarely in angiosperms.

## 5. Conclusion

Because siring success depends largely on the total number of pollen grains exported to receptive stigmas, both pollen removal and successful transport of removed pollen are key fitness components for plants. Neither of these two components alone provides a complete understanding of the pollen economy and its ecological and evolutionary consequences. This study identified packaging of pollen into different dispersal units as a key trait accounting for the marked differences in pollen economy observed among plant species and showed that among species with the same dispersal type, pollen economy is not strongly predicted by the diversity or functional type of pollinators.

## Data Availability

All data analysed in this study are included in the manuscript and supporting data table file. All software packages used for analysis (in particular, statistical analysis) are cited in the methods. The specific code used to implement the statistical analyses is provided in the electronic supplementary material, appendices A3 and A4 [100].
